# Resveratrol Alleviates Hepatic Fibrosis in Associated with Decreased Endoplasmic Reticulum Stress-Mediated Apoptosis and Inflammation

**DOI:** 10.1007/s10753-021-01586-w

**Published:** 2022-01-26

**Authors:** Zhenyu Ma, Lulu Sheng, Juan Li, Jianmin Qian, Gang Wu, Zhengxin Wang, Yi Zhang

**Affiliations:** 1 Department of General Surgery, Huashan Hospital, Fudan University, Shanghai, 200040 China; 2 Department of Emergency Medicine, Shanghai Jiao Tong University Affiliated Sixth People’s Hospital, Shanghai, 200233 China; 3 Department of Nursing Center, Huashan Hospital, Fudan University, Shanghai, 200040 China; 4 Biomedical Research Center, Institute for Clinical Sciences, Zhongshan Hospital, Fudan University, Shanghai, 200032 China; 5 Shanghai Key Laboratory of Organ Transplantation, Shanghai, China

**Keywords:** hepatic fibrosis, resveratrol, endoplasmic reticulum stress, inflammation

## Abstract

Hepatic fibrosis (HF) is the typical response to chronic liver disease and is characterized by deposition of abundant extracellular matrix. The aim of the present study was to investigate the protective effect of resveratrol (RSV) in a CCl_4_-induced rat model of HF. We demonstrate that the administration of RSV effectively improves liver function and ameliorates liver fibrosis by reducing collagen deposition and reversing the expression of COL1A1 and PPAR-γ. Treatment efficacy of RSV could be attributed to reversed epithelial-mesenchymal transition progress with upregulated expression of E-cadherin and downregulated N-cadherin, vimentin, and α-SMA. Moreover, RSV significantly decreased the levels of endoplasmic reticulum stress (ERS)-related proteins CHOP; Bip; cleaved caspase-3, caspase-7, and caspase-12; Bax; and Bak while promotes the expression of anti-apoptosis protein Bcl2. The important role of ERS in HF was confirmed by using 4-PBA, an ERS inhibitor, which markedly ameliorated CCl_4_-induced HF. Further, mechanistic studies demonstrated that RSV significantly decreased CCl_4_-induced transforming growth factor-β synthesis and inflammatory factor (tumor necrosis factor-α and interleukin-6) expression and reduced the inflammation of hepatic stellate cells by inhibiting the NF-κB pathway *in vivo* and *in vitro*. In conclusion, the results suggested that RSV ameliorated HF in associated with decreased ERS-induced apoptosis and inflammation in rats.

**Supplementary Information:**

The online version contains supplementary material available at 10.1007/s10753-021-01586-w.

## INTRODUCTION

Hepatic fibrosis (HF) is a serious health problem all over the world, contributing to the pathological progression of chronic liver disease to cirrhosis and cancer. Currently, no effective therapy was found to completely reverse the disease, while interventions from the early stage may largely block the progression of HF [1]. With the stimulation of tissue injury, quiescent hepatic stellate cells (HSCs) are activated and transdifferentiate into myofibroblasts, which are responsible for the excessive deposition of extracellular matrix (ECM), especially interstitial collagen [2]. HSCs are considered to be the main target of a number of profibrotic factors, including transforming growth factor (TGF)-β [3].

The endoplasmic reticulum (ER) is the intracellular organelle responsible for the synthesis, folding, trafficking, and maturation of proteins. The ER interacts with other intracellular organelles, including mitochondria, the Golgi apparatus, endosomes, peroxisomes, and the plasma membrane [4]. The liver synthesizes a large number of proteins and lipids, and hepatocytes have well-functioning ERs with appropriate adaptive capability. Pathological stimuli, including inflammation and oxidative stress, can disrupt the function of the ER, resulting in the aggregation of misfolded proteins in the ER cavity, which causes cell homeostasis imbalance and hepatic dysfunction [5]. Preclinical studies have suggested that upregulation of ER stress (ERS)-related proteins, including binding immunoglobulin protein (BIP), Bcl-2 associated X protein (Bax), Bcl-2 homologous antagonist/killer (Bak), and C/EBP homologous protein (CHOP), occurs in animal models of HF [6, 7]. CHOP may lead to HF by increasing the expression of fibrotropic factors, including α-smooth muscle actin (α-SMA), TGF-β, and collagen [8]. In addition, a number of studies have reported that ERS promotes HF progression by activating HSCs [9, 10]. In a CCl_4_-induced rat model of HF, the protein and mRNA expression levels of Tribbles homolog 3 (TRB3) and CHOP were upregulated, which suggested that ERS may induce hepatocytes apoptosis via TRB3 and CHOP [11].

Resveratrol (RSV) is a natural polyphenolic compound, containing an Astragalus membranaceus structure and non-flavonoids, and is primarily found in red wine and peanuts. Recently, it has been reported that RSV displays antitumor, antioxidation, anti-inflammatory, hypoglycemic, analgesic, and antiasthmatic effects [12]. It has also been reported that RSV displays protective effects against cardiovascular [13] and liver disease including CCl_4_-induced HF [14], but the mechanisms remain unclear. In the present study, the specific mechanisms underlying the effects of RSV on HF, and whether RSV inhibits ERS-mediated apoptosis and inflammation in a rat model of HF were investigated.

## MATERIALS AND METHODS

### Animals

Sprague–Dawley male rats (age, 3–4 weeks; weight, 200–250 g) were purchased from Shanghai SLAC Experimental Animal Co., Ltd. Animals were housed at 22 ± 1 °C, with 50 ± 1% humidity, 12 h light/dark cycles, and free access to water and food (standard diet). The present study was approved by the Bioethics Committee of Huashan Hospital, Fudan University, Shanghai.

### HF Model Establishment and Primary HSC Isolation

Rats were anaesthetized by intraperitoneal injection of 0.1% sodium pentobarbital (40 mg/kg). Subsequently, the rats were subcutaneously injected with 40% CCl_4_ (Beijing Beihua Fine Chemicals Co., China) dissolved in olive oil suspension (Beyotime Biotechnology, Jiangsu Province, China; 2 ml/kg twice a week) and intragastrically administer with 0.5% carboxymethylcellulose (CMC) sodium salt (Sigma-Aldrich, St. Louis, MO) once a day for 12 weeks with or without RSV (Sigma-Aldrich) daily treatment intragastrically for 12 weeks. The control group was treated olive oil and was orally administered sodium CMC. In some experiments, the ERS inhibitor, 4-PBA (Sigma-Aldrich), was intraperitoneally administrated (10 mg/kg). At the end of the 12 weeks, rats were anesthetized with 40 mg/kg sodium pentobarbital, and 5–10 ml blood was collected from the heart; following blood collection, rats were euthanized by cervical dislocation, and liver tissue samples were collected. To isolate HSCs in the indicated time, livers were digested by collagenase/pronase. The HSCs were obtained from the non-parenchymal cell fraction by density-gradient centrifugation using 28% Nycodenz (Nycomed Pharma, Oslo, Norway) and fluorescence-activated cell sorting using their typical retinoid fluorescence.

### Liver Function Analysis

The serum of rats was collected to measure the levels of alanine aminotransferase (ALT) and aspartate aminotransferase (AST) using a biochemical analyzer (Hitachi, Japan); alkaline phosphatase (ALP) and γ-glutamyl transpeptidase (GGT) were assessed using commercial kits purchased from Westang Company (Shanghai, China).

### Cell Culture and Treatment

The immortalized human HSC cell line LX-2 (provided by Department of Liver Surgery and Transplantation, Liver Cancer Institute, Zhongshan Hospital, Fudan University) were routinely cultured in DMEM medium containing 10% fatal bovine serum. Cells were treated with or without various concentrations of RSV and stimulated with TGF-β for 24 h.

### Histological Examination

Liver tissues were fixed, embedded in paraffin, and cut into 5-μm-thick sections. Subsequently, sections were dewaxed and stained with Hematoxylin & Eosin (H&E), Sirius red, or Masson’s trichrome. Severity of liver steatosis was determined according to the 2006 criteria of the Chinese Medical Association Committee of Fatty Liver Disease and Nouchi et al. [15, 16]. Steatosis was graded based on the extent of parenchyma involved: grade 0, no hepatocytes involved; grade 1, < 30% of hepatocytes involved; grade 2, 30–50% of hepatocytes involved; grade 3, 51–75% of hepatocytes involved; and grade 4, > 75% of hepatocytes involved. The stage of HF was graded using the METAVIR scale [17], which grades fibrosis on a 5-point scale: grade 0, no fibrosis; grade 1, portal fibrosis without septa; grade 2, portal fibrosis with a few septa; grade 3, numerous septa without cirrhosis; and grade 4, cirrhosis. For the quantification of collagen deposition in Sirius red or Masson’s trichrome staining, six different high power fields from each section were randomly chosen and examined using computer-assisted image analysis. The optical density and color area percentage of collagen fibers were assessed. Sections were observed under a light microscope and analyzed by a pathologist who was blinded to the different treatments.

### Quantitative PCR (qPCR)

Total RNA was extracted from liver tissues using TRIzol® reagent (Thermo Fisher Scientific, Bremen, Germany). Total RNA (0.5 μg) in 10 μl volume was reverse transcribed into cDNA using the RevertAid™ First Strand cDNA Synthesis kit (Thermo Fisher Scientific) according to the manufacturer’s protocol. The reverse transcription steps included 37 °C for 15 min, 85 °C for 5 min, and a termination step at 4 °C. qPCR was performed using the SYBR Premix Ex Taq kit (Takara Bio, Shiga, Japan) and the applied biosystem (Thermo Fisher Scientific) according to the manufacturer’s protocol. The primer pairs used for qPCR are presented in Table 1. The following thermocycling conditions were used for qPCR: after initiation (30 s at 95 °C), amplification was performed for 45 cycles including denaturation (5 s at 95 °C), annealing (30 s at 55 °C), and extension (60 s at 72 °C). mRNA expression levels were quantified using the 2 − ΔΔCq method and normalized to the internal reference gene GAPDH.

**Table 1:** qPCR Primers

Name	Sequence
GAPDH forward	5′-AGGTCGGTGTGAACGGATTT-3′
GAPDH reverse	5′-GGGGTCGTTGATGGCAACA-3′
TGF-β forward	5′-CTTTGTACAACAGCACCCGC-3 ′
TGF-β reverse	5′-CGGGTGACTTCTTTGGCGTA-3′
COL1A1 forward	5′-TGACGCATGGCCAAGAAGAC-3 ′
COL1A1 reverse	5′-TCTTTGCATAGCACGCCATC-3′
TNF-α forward	5′-ATGAGCACAGAAAGCATGATG-3′
TNF-α reverse	5′-TACAGGCTTGTCACTCGAATT-3′
PPAR-γ forward	5′-ATTCTGGCCCACCAACTTCGG-3′
PPAR-γ reverse	5′-TGGAAGCCTGATGCTTTATCCCCA-3 ′
IL-6 forward	5′-CCGGAGAGGAGACTTCACAG-3′
IL-6 reverse	5′-ACAGTGCATCATCGCTGTTC-3′

### ELISA

Liver tissue samples were analyzed using the Westang ELISA kit according to the manufacturer’s protocol. Briefly, the tissue lysate supernatant and standard were transferred to an ELISA plate and mixed for 40 min at 37 °C. After washing, the first anti-working liquid, enzyme-labeled antibody working liquid, and substrate working liquid were added to each well. Subsequently, the reaction was terminated using a termination solution, and the absorbance of each well was measured at a wavelength of 450 nm. Sample contents were determined using an ELISA standard curve, which consisted of the concentration gradient of the standard substance on the *x* axis plotted against the optical density value on the *y* axis.

### Western Blot

Liver tissues or cells were lysed, and total protein was quantified using a bicinchoninic acid assay (Beyotime Biotechnology). Proteins (20 µg) were separated via 12% SDS‑PAGE and transferred onto polyvinylidene difluoride membranes at 300 mA for 120 min. The membranes were blocked with 5% BSA (Beyotime Biotechnology) for 1 h at room temperature. Subsequently, the membranes were incubated overnight at 4 °C with the following primary antibodies diluted 1,000-fold that recognized COL1A1; PPAR-γ; E‑cadherin; N-cadherin; vimentin; α-SMA; CHOP; BIP; cleaved caspase-3, caspase-7, and caspase-12; Bcl2; Bax; Bak; p-p65-NF-κB; p65-NF-κB; p-IκBα; IκBα; and β-actin (all from Cell Signaling, USA or Abcam, UK). Following primary incubation, the membranes were washed with TBS Tween 20 (1%) and incubated with a horseradish peroxidase‑conjugated goat anti‑rabbit secondary antibody (1:10,000) at room temperature for 1 h. Protein bands were visualized using an enhanced chemiluminescence system (Thermo Fisher Scientific). Protein band intensities were examined using Image-Pro Plus software (version 6.0; Media Cybernetics, MD, USA), and all values were normalized to β-actin. Data of liver tissues and cultured cells are calculated from three independent experiments.

### Statistical Analysis

Data are expressed as the mean ± standard deviation (SD) and analyzed by SPSS 13.0 (SPSS, Chicago, IL, USA). Survival analysis was performed using the Kaplan–Meier method, and survival curves were compared using Bonferroni’s test. Comparisons between two groups were analyzed using the Student’s *t*-test. Multiple comparisons were analyzed using one-way ANOVA followed by Tukey’s post hoc test. A *p* < 0.05 was considered to indicate a statistically significant difference.

## RESULTS

### RSV Effectively Decreases the Liver Function Index in a CCl_4_-Induced Rat Model of HF

**Fig. 1:**
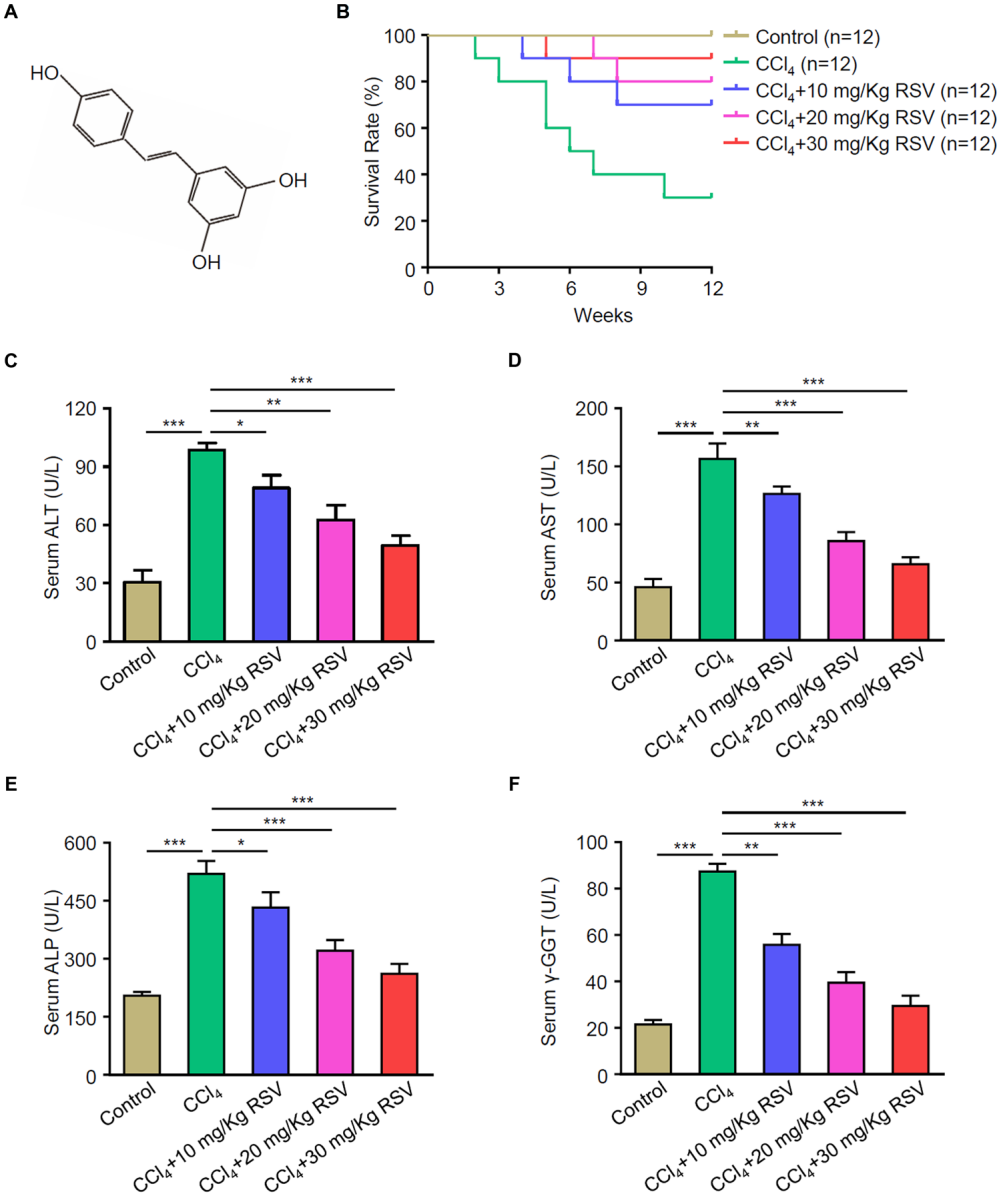
Effect of RSV on liver function. Rat HF models were established with or without RSV treatment. Peripheral blood were collected after 3 months. **A** The chemical structure of RSV. **B** The survival rate of rats were analyzed (*n* = 12). Serum levels of ALT (**C**), AST (**D**), ALP (**E**), and γ-GGT (**F**) were tested using commercial kits. Data are mean ± SD from three independent experiments (*n* = 6 rats per group). For the high mortality of CCl_4_-induced HF and the average survival rate is about 25%, more than 12 rats were used for each experiment to make sure that at least 3 rats were survived in the CCl_4_ group 3 months after HF development. *p < 0.05, **p < 0.01, and ***p < 0.001.

The chemical structure of RSV is presented in Fig. 1A. The results indicated that RSV significantly increased the survival rate in the rat of CCl_4_ group (Fig. 1B). To further explore the protective effect of RSV on the liver, liver function indices, including ALT, AST, ALP, and γ-GGT, in each group were measured. Compared with the control group, liver function indices were significantly increased in the CCl_4_ group, and RSV treatment, particularly with a dose of 30 mg/kg, significantly reversed CCl_4_-induced effects on liver function (Fig. 1C-F).

### RSV Reduces CCl_4_-Induced Fat and Collagen Deposition and Ameliorates CCl_4_-Induced Liver Fibrosis

**Fig. 2:**
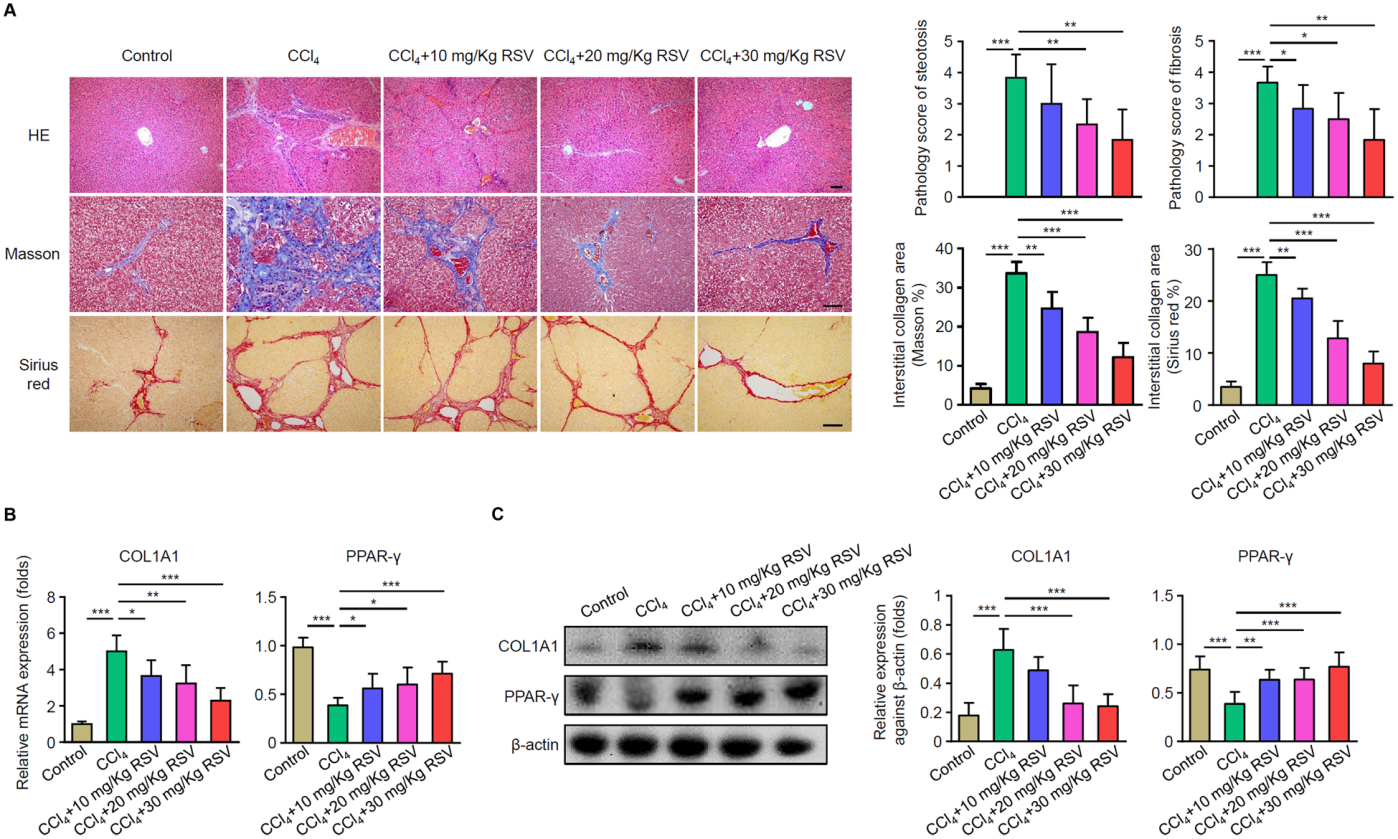
RSV alleviates the CCl_4_-induced rat model of hepatic fibrosis. Rat HF models were established with or without RSV treatment. Liver tissues were collected after 3 months. **A** Livers were sectioned for H&E, Masson’s trichrome, and Sirius red staining in the treatment groups. Representative images were shown on the left (scale bars, 40 μm) and summarized results are on the right. **B** mRNA levels of COL1A1 and PPAR-γ in livers were measured by qPCR. Data are normalized to expression levels of control. **C** Protein levels of COL1A1 and PPAR-γ in livers were measured by western blot. Representative images were shown on the left and summarized results are on the right. **A–C** Data are mean ± SD from three independent experiments (*n* = 6 rats per group). More than 12 rats were used for each experiment to make sure that at least 3 rats were survived in the CCl_4_ group 3 months after HF development. *p < 0 .05, **p < 0.01, and ***p < 0.001.

Pathological analysis was performed to evaluate the degree of hepatic injury and fibrosis induced by CCl_4_ in rats. The structure of hepatic lobules was disordered in the CCl_4_ group, which was indicated by hepatocyte steatosis, necrosis, inflammatory cell infiltration, hepatic lobule separation by collagen fibers, and a large amount of collagen fiber deposition in the portal area. RSV treatment restored the hepatic lobule structure, necrosis, inflammatory cell infiltration, and fibrous tissue proliferation in the portal canal area (Fig. 2A). Moreover, the 10, 20, and 30 mg/kg of RSV significantly decreased the interstitial collagen area dose dependently using Masson’s trichrome and Sirius red staining (Fig. 2A). To further study role of RSV on liver fibrosis, we then evaluated the levels of COL1A1 and PPAR-γ in livers. As shown in Fig. 2B and C, RSV treatment significantly reversed the expression of COL1A1 and PPAR-γ in both mRNA and protein levels. These results suggested that RSV reduces CCl_4_-induced collagen deposition and liver fibrosis.

### RSV Reduces CCl_4_-Induced Epithelial-Mesenchymal Transition (EMT) and ERS in Rats

**Fig. 3:**
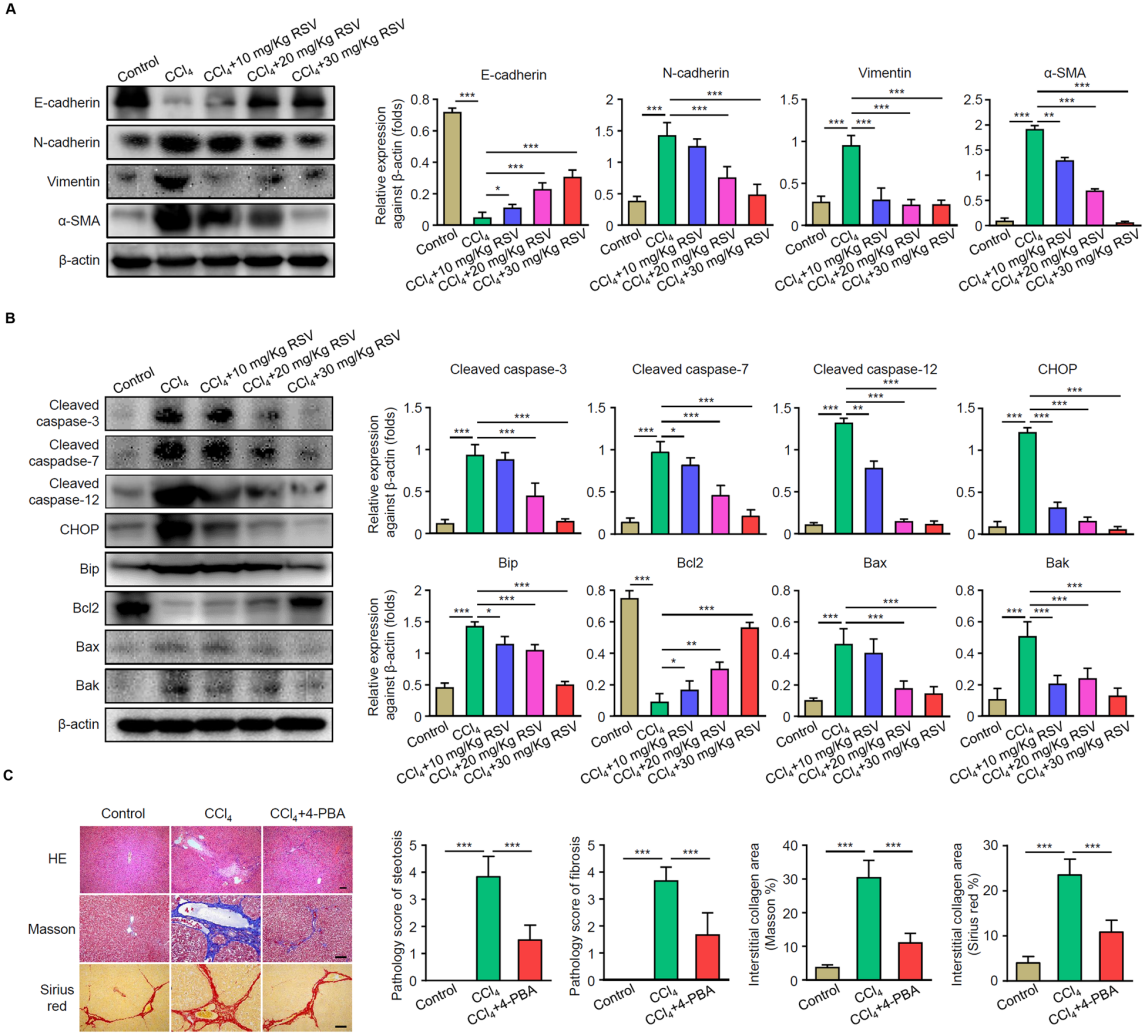
RSV significantly reduces CCl_4_-induced EMT and ERS. Rat HF models were established with or without RSV treatment. Liver tissues were collected after 3 months. **A** Protein levels of EMT-related markers E-cadherin, N-cadherin, vimentin, and α-SMA were determined by western blot. **B** Protein levels of ERS and apoptosis-related proteins CHOP; BIP; cleaved caspase-3, caspase-7, and caspase-12; Bcl2; Bax; and Bak were determined by western blot. **C** HF rats were administered with 4-PBA, and livers were sectioned for H&E, Masson’s trichrome, and Sirius red staining in each group. **A–C** Representative images were shown on the left and summarized results from three independent experiments are shown as mean ± SD on the right (*n* = 6 rats per group). More than 12 rats were used for each experiment to make sure that at least 3 rats were survived in the CCl_4_ group 3 months after HF development. *p < 0.05, **p < 0.01, and ***p < 0.001.

EMT and ERS are associated with HF. Western blotting was performed to measure the expression levels of EMT- and ERS-related proteins. The expression of E-cadherin (epithelial marker) was decreased, whereas levels of N-cadherin, vimentin, and α-SMA (mesenchymal markers) were increased in the CCl_4_ group; RSV treatment significantly reversed their expression (Fig. 3A). In addition, RSV treatment largely decreased the levels of ERS-related proteins CHOP and BIP, and apoptosis-related proteins cleaved caspase 3, 7, 12, Bax and Bak, whereas promoted the expression of anti-apoptosis protein Bcl2. To further investigate whether ERS participated in CCl_4_-induced HF, 4-PBA (an ERS inhibitor) was administrated by intraperitoneal injection. As shown in Fig. 3C, pathological analysis indicated that 4-PBA markedly ameliorated the structure of hepatic lobules, with low levels of necrosis, inflammatory cell infiltration, and collagen deposition in the liver of CCl_4_ treated rats. The results indicated that RSV reduces CCl_4_-induced EMT and ERS.

### RSV Significantly Reduces CCl_4_-Induced TGF-β Synthesis and Inflammatory Factor Expression

**Fig. 4:**
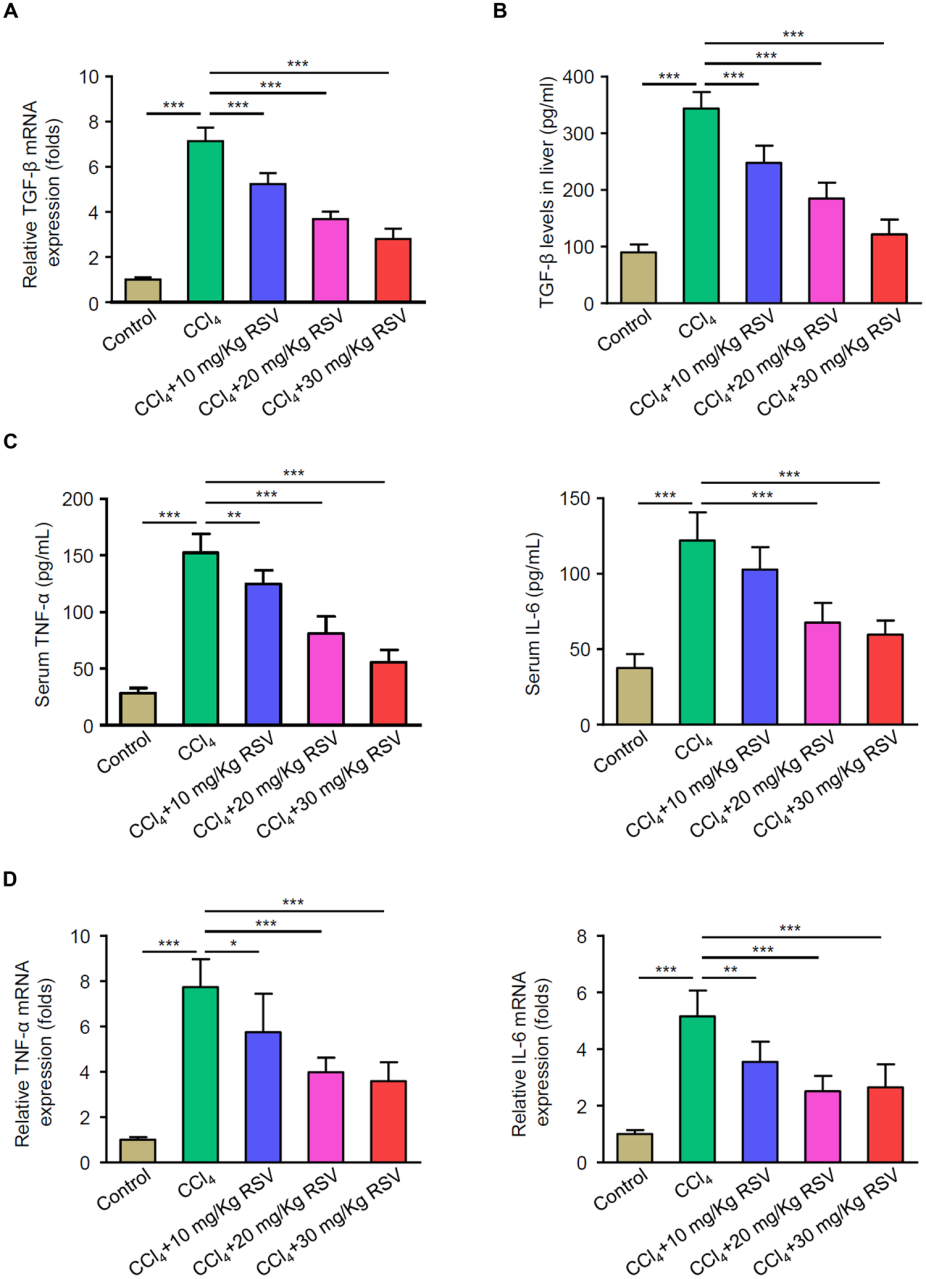
Effect of RSV on CCl_4_-induced TGF-β synthesis and inflammatory factor expression. Rat HF models were established with or without RSV treatment. Peripheral blood and liver tissues were collected after 3 months. HSCs were isolated from livers 1 month after establishment. **A** mRNA expression levels of TGF-β in liver tissue were measured by qPCR. **B** Secretory TGF-β levels in livers were measured by ELISA. **C** Serum levels of inflammatory factors TNF-α and IL-6 were measured by ELISA. **D** mRNA levels of TNF-α and IL-6 in liver HSCs were measured by qPCR. **A–D** Results from three independent experiments are shown as mean ± SD (*n* = 6 rats per group). More than 12 rats were used for each experiment to make sure that at least 3 rats were survived in the CCl_4_ group 3 months after HF development. *p < 0.05, **p < 0.01, and ***p < 0.001.

To investigate the involvement of TGF-β during HF, the expression levels of TGF-β in the liver were detected using ELISA and qPCR. Secretion and mRNA levels of TGF-β in the liver were significantly increased in the CCl_4_ group compared with the control group, and RSV treatment decreased CCl_4_-induced TGF-β levels, which was significantly attenuated by 30 mg/kg RSV (Fig. 4A and B). Furthermore, serum levels of inflammatory factors tumor necrosis factor (TNF)-α and interleukin (IL)-6 were reversed by RSV treatment (Fig. 4C). As HSC activation plays a vital role in HF, we isolated liver HSCs 1 month after CCl_4_ induction in each group and found that RSV treatment greatly decreased the mRNA levels of TNF-α and IL-6 in HSCs as compared with that of CCl_4_-treated rats (Fig. 4D). These data demonstrated that RSV significantly reduces CCl_4_-induced TGF-β synthesis and inflammatory factor expression in livers and HSCs.

### RSV Significantly Reduces the Inflammation of HSCs by Inhibiting the NF-κB Pathway *In Vitro*

**Fig. 5:**
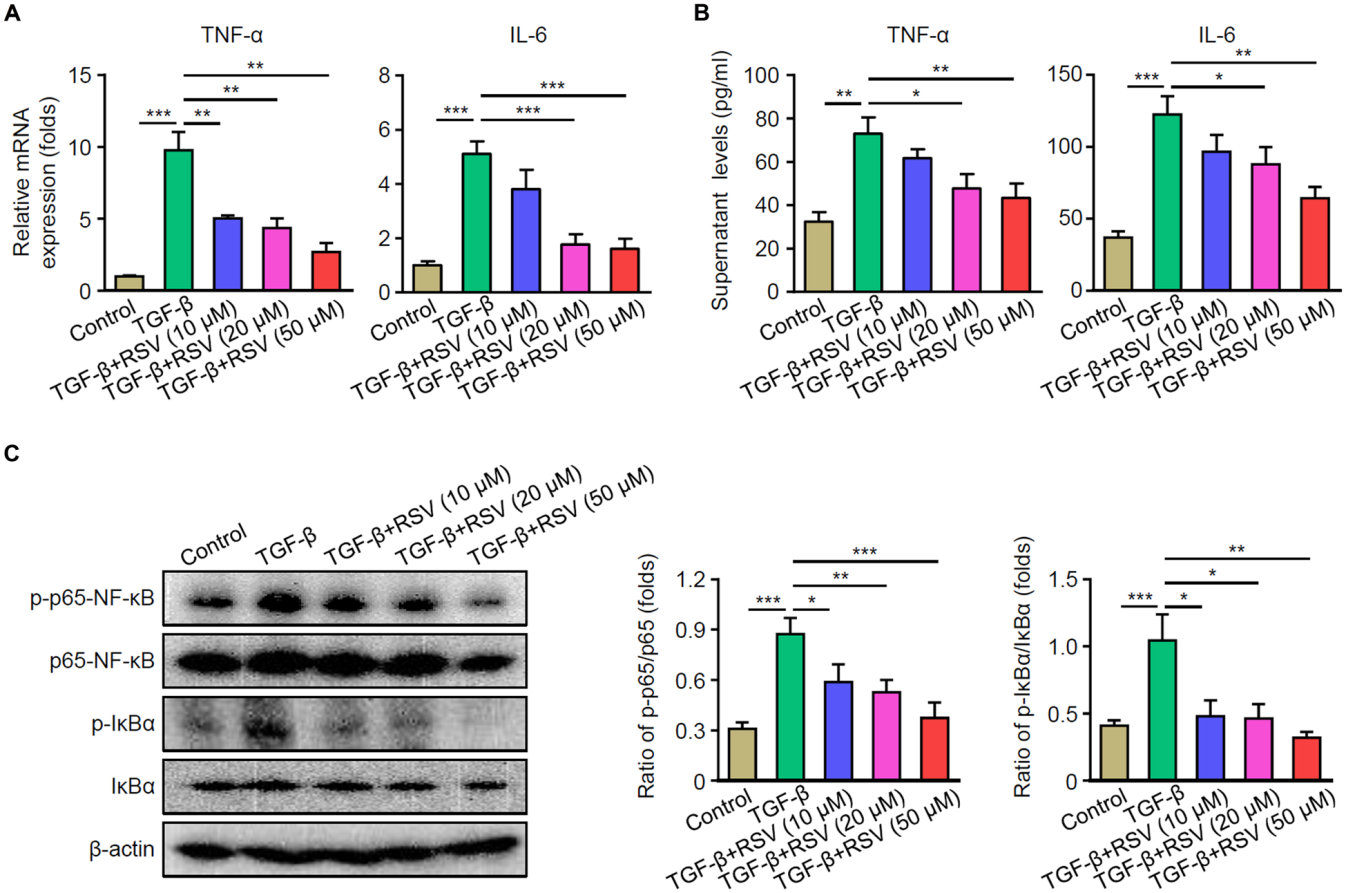
RSV reduces LX-2 cell inflammation by inhibiting the NF-κB pathway *in vitro*. LX-2 cells were treated with or without RSV (10, 20, or 50 µM) and stimulated with TGF-β for 24 h. mRNA levels and supernatant levels of TNF-α and IL-6 were measured by qPCR (**A**) and ELISA (**B**). **C** Levels of p-p65-NF-κB, p65-NF-κB, p-IκBα, IκBα, and β-actin were measured by western blot. Representative images were shown on the left and summarized results are on the right. **A–C** Results from three independent experiments are shown as mean ± SD (*n* = 3). *p < 0.05, **p < 0.01, and ***p < 0.001.

To further dissect the cellular mechanisms involved in the protective role of RSV in HSCs, we set out to induce the activation of human HSC cell line LX-2 with TGF-β *in vitro* for 24 h. As shown in Fig. 5A and B, mRNA levels in HSCs and secretion levels in the supernatant of TNF-α and IL-6 after TGF-β induction were greatly increased, whereas RSV treatment significantly decreased the levels of inflammatory factors. Moreover, RSV treatments abolished the phosphorylation of p65- NF-κB and IκBα in a dose-dependent manner (Fig. 5C). Thus, RSV significantly reduces the HSC inflammation by inhibiting the NF-κB pathway *in vitro*.

## DISCUSSION

HF is the most common pathological feature of chronic liver disease; however, a limited number of effective drugs are available within clinics [18]. RSV is a polyphenolic compound and has been used for the treatment of atherosclerosis, as well as cardiovascular and cerebrovascular diseases [13]. Furthermore, previous studies have reported that RSV effectively inhibits HF in rats; however, the specific mechanism of action and effective dosage has not been identified [19, 20]. In the present study, rats were administered with CCl_4_ and different concentrations of RSV. After 12 weeks, liver function was measured to evaluate the degree of liver damage. The results indicated that compared with the control group, the liver function was severely impaired in the CCl_4_ group, which was characterized by degeneration and necrosis of hepatocytes, and abnormal deposition of collagen. Following treatment with RSV, the liver function was significantly improved, and the serum indices, including ALT, AST, ALP, and γ-GGT, were significantly decreased. Furthermore, the degeneration and necrosis of hepatocytes were significantly reduced, suggesting that RSV may inhibit HF progression.

Recent studies have demonstrated that HSC activation is essential in the pathogenesis of liver fibrosis and abnormal activated EMT is suggested to potentially promote HSC activation [21]. In the present study, the expression of E-cadherin (epithelial marker) in the CCl_4_ group was downregulated compared with the control group, while the expression of N-cadherin, vimentin, and α-SMA (mesenchymal markers) was upregulated. The results indicated that the HSCs may undergo EMT, which was demonstrated by the loss of epithelial markers and the gain of mesenchymal markers, to transform into fibroblasts or mesenchymal cells following CCl_4_ stimulation. However, treatment with RSV increased the expression of E-cadherin and decreased the expression of N-cadherin, vimentin, and α-SMA compared with the CCl_4_ group, which indicated that RSV could reduce CCl_4_-induced EMT.

Caspases participate ERS-induced apoptosis. Previous studies have reported that the caspase-12 signaling pathway is closely associated with the development of cholestatic HF and non-alcoholic HF [22, 23]. In response to ERS, caspase-7 rapidly cleaves and activates caspase-12, leading to the activation of downstream caspases, including caspase-3 [24]. Importantly, the balance of proapoptotic Bax, Bak, and antiapoptotic Bcl-2 proteins in mitochondria determine the fate of cells. Tian et al. demonstrated that the protein expression of Bcl-2 in the liver tissue was downregulated, while levels of Bak and Bax were upregulated in rats with CCl_4_ stimulation [7]. In the present study, RSV treatment largely decreased the expression levels of ERS-related proteins CHOP; BIP; cleaved caspase-3, caspase-7, and caspase-12; Bax; and Bak while promotes the expression of anti-apoptosis protein Bcl2, indicating that RSV is protective in ERS-induced apoptosis.

The occurrence and development of HF are regulated by different cytokines, including TGF-β and NF-κB [22]. Unfolded protein response-induced apoptosis can lead to TGF-β secretion, which in turn promotes HF [25]. ERS participates in HF by regulating the release of inflammatory cytokines such as TNF-α and IL-6, which are the main factors inducing HF [26, 27]. Moreover, the TGF-β signaling pathway not only serves a key role in the HF process, but is also involved in the activation and transformation of HSCs [28]. Wang et al. recently indicated that TGF-β-mediated NF-κB pathway plans an important role in HSC activation and HF both *in vivo* and *in vitro*, which can be restored by treatment of the active components of *Schisandra chinensis*, a traditional Chinese medicine [29]. The present study suggested that RSV inhibited the release of TGF-β and subsequent systemic and HSC inflammation in associated with decreased ERS-induced apoptosis and inflammation via NF-κB dependent manner in HF rats.

In conclusion, our findings demonstrate that RSV effectively ameliorates the severity of CCl_4_-induced HF in associated with the decreased EMT and ERS in livers. Mechanistically, treatment of RSV inhibits the systemic and HSC inflammation in a NF-κB dependent manner. Currently, we are still working on optimizing the treatment of RSV in CCL4-induced liver fibrosis, and we believe the work will be benefit to disclose the protective window of time of RSV and clarify the molecular and cellular mechanisms in future.

## Supplementary Information

Below is the link to the electronic supplementary material.

Supplementary file1 (PNG 408 KB)

Supplementary file2 (PNG 496 KB)

Supplementary file3 (PNG 516 KB)

Supplementary file4 (PNG 387 KB)

Supplementary file5 (PNG 643 KB)

Supplementary file6 (PNG 584 KB)

Supplementary file7 (PNG 515 KB)

Supplementary file8 (PNG 431 KB)

Supplementary file9 (PNG 479 KB)

Supplementary file10 (PNG 1527 KB)

Supplementary file11 (PNG 1527 KB)

Supplementary file12 (PNG 588 KB)

Supplementary file13 (PNG 536 KB)

Supplementary file14 (PNG 1527 KB)

## Data Availability

All data used during the study are available through contacting the corresponding author by request.
