# Supplementary figures and images for: Resveratrol Alleviates Hepatic Fibrosis in Associated with Decreased Endoplasmic Reticulum Stress-Mediated Apoptosis and Inflammation
Source: Inflammation. 2022 Jan 26;45(2):812–23. doi: 10.1007/s10753-021-01586-w (PMC8956545; doi:10.1007/s10753-021-01586-w)

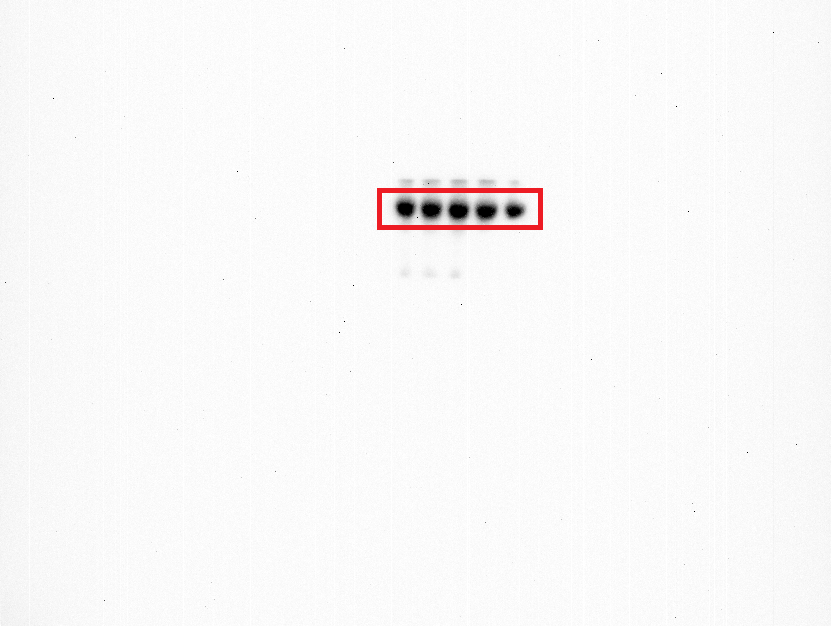

Supplement: Supplementary file 1 — Supplementary file1 (PNG 408 KB) [file 10753_2021_1586_MOESM1_ESM.png]

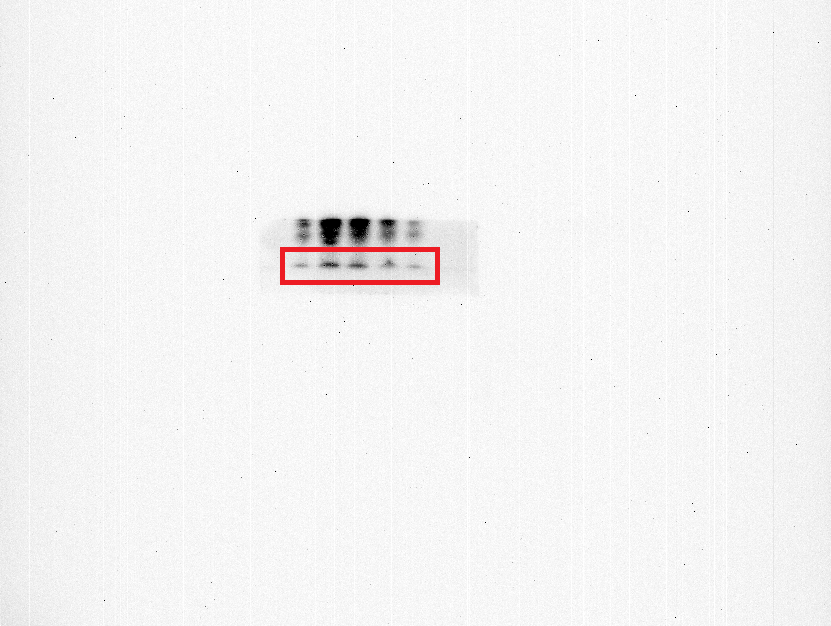

Supplement: Supplementary file 2 — Supplementary file2 (PNG 496 KB) [file 10753_2021_1586_MOESM2_ESM.png]

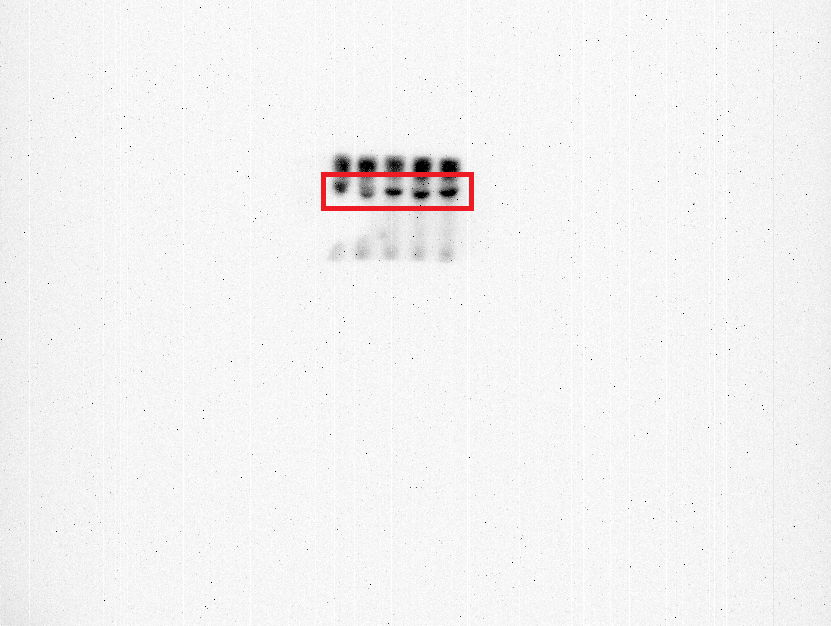

Supplement: Supplementary file 3 — Supplementary file3 (PNG 516 KB) [file 10753_2021_1586_MOESM3_ESM.png]

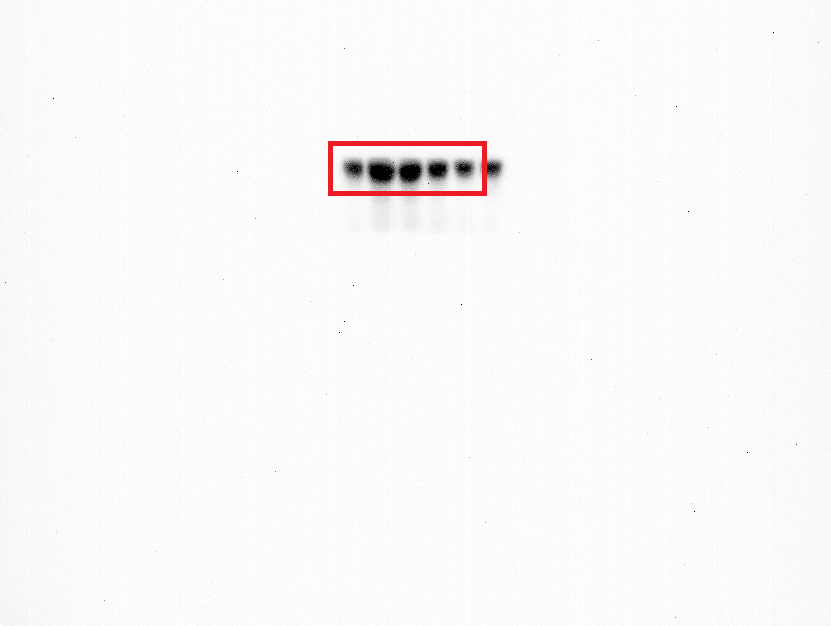

Supplement: Supplementary file 4 — Supplementary file4 (PNG 387 KB) [file 10753_2021_1586_MOESM4_ESM.png]

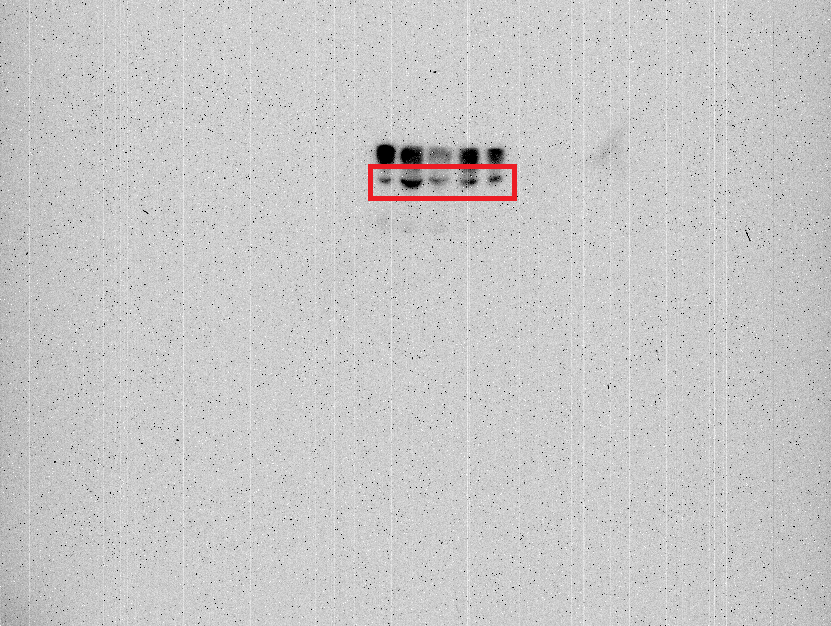

Supplement: Supplementary file 5 — Supplementary file5 (PNG 643 KB) [file 10753_2021_1586_MOESM5_ESM.png]

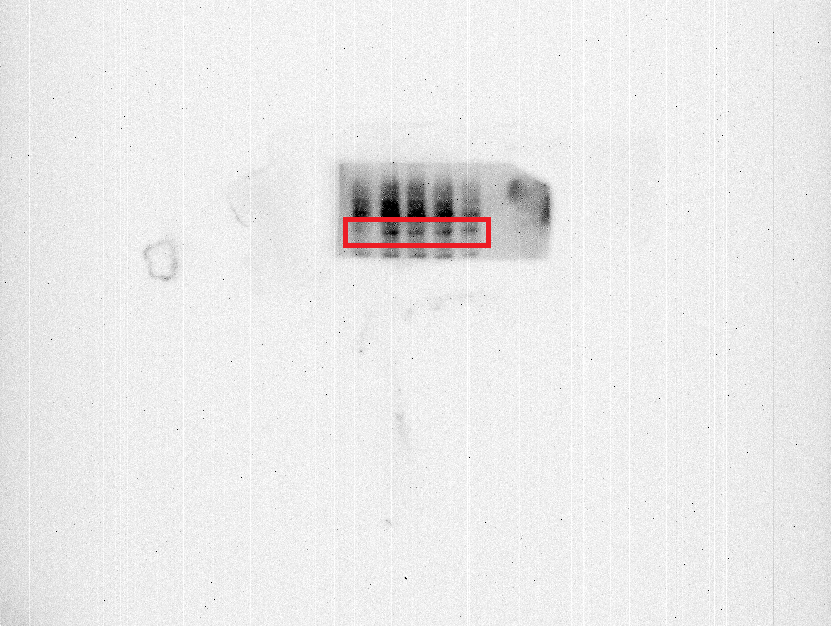

Supplement: Supplementary file 6 — Supplementary file6 (PNG 584 KB) [file 10753_2021_1586_MOESM6_ESM.png]

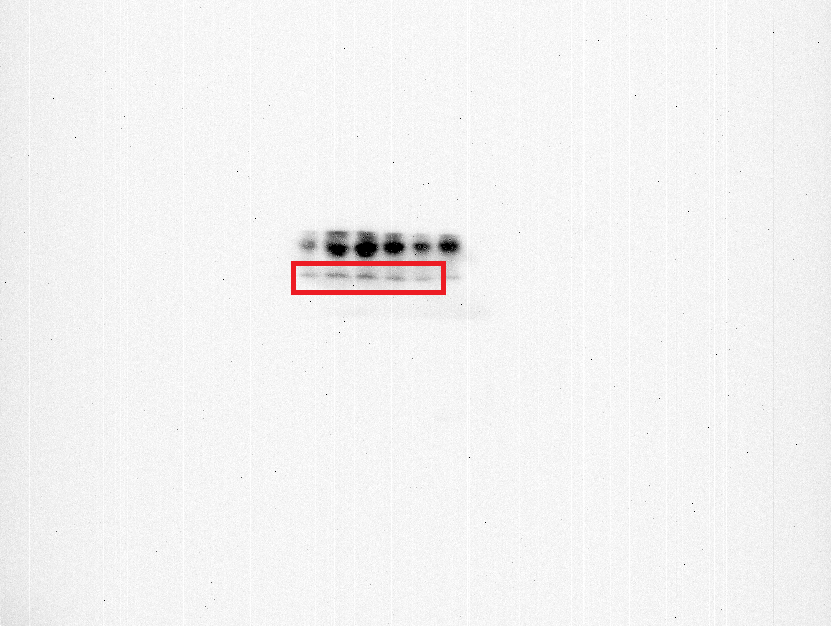

Supplement: Supplementary file 7 — Supplementary file7 (PNG 515 KB) [file 10753_2021_1586_MOESM7_ESM.png]

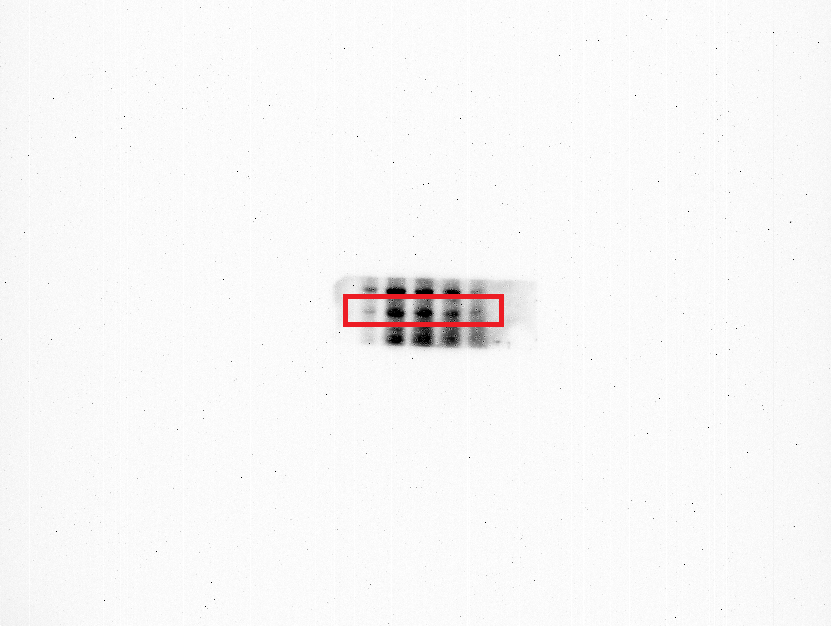

Supplement: Supplementary file 8 — Supplementary file8 (PNG 431 KB) [file 10753_2021_1586_MOESM8_ESM.png]

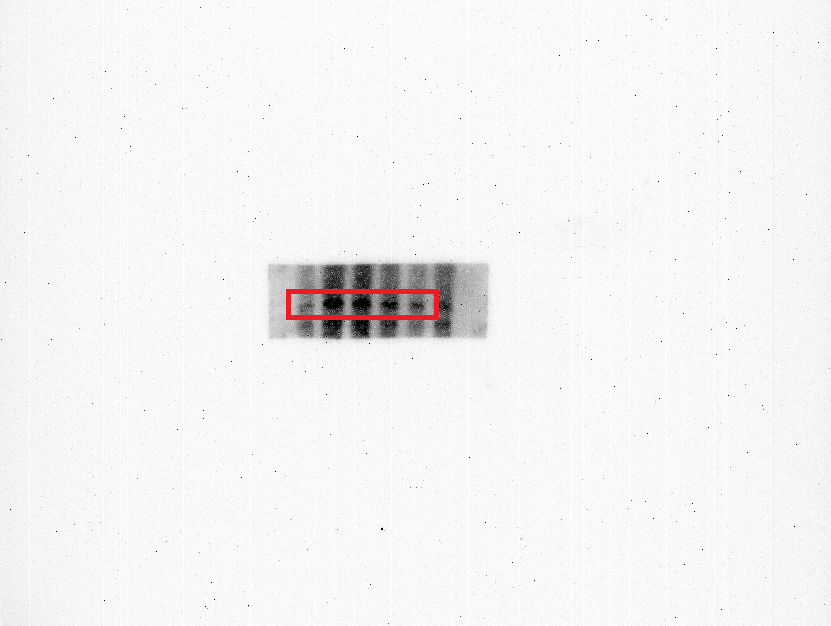

Supplement: Supplementary file 9 — Supplementary file9 (PNG 479 KB) [file 10753_2021_1586_MOESM9_ESM.png]

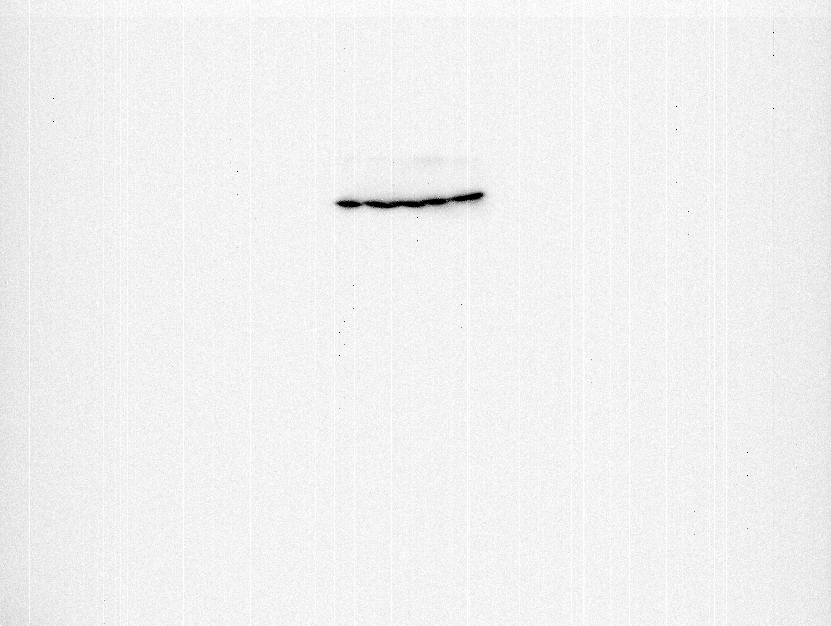

Supplement: Supplementary file 10 — Supplementary file10 (PNG 1527 KB) [file 10753_2021_1586_MOESM10_ESM.png]

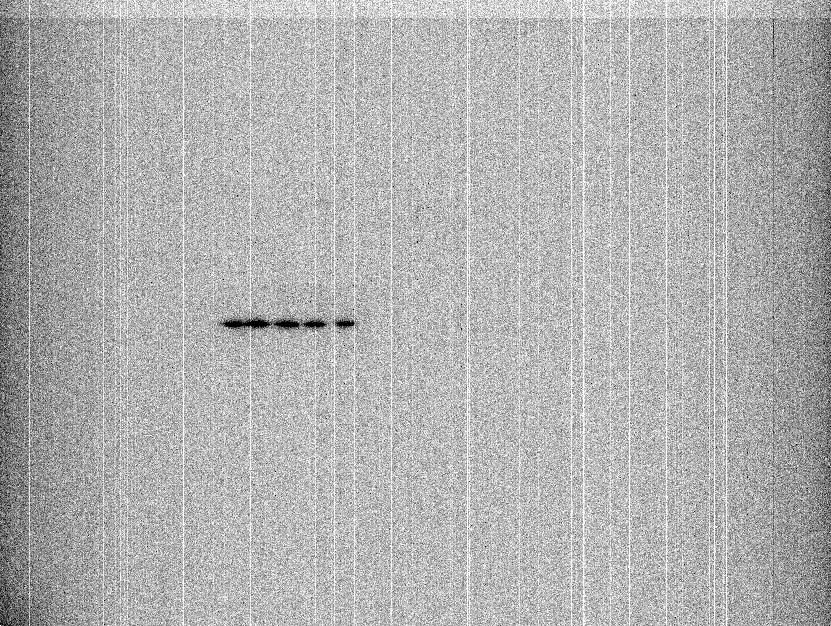

Supplement: Supplementary file 11 — Supplementary file11 (PNG 1527 KB) [file 10753_2021_1586_MOESM11_ESM.png]

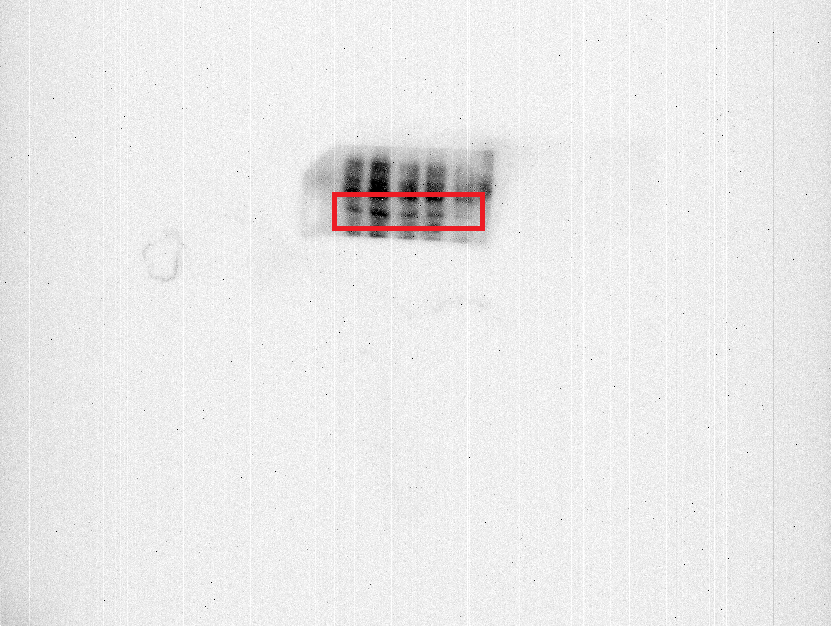

Supplement: Supplementary file 12 — Supplementary file12 (PNG 588 KB) [file 10753_2021_1586_MOESM12_ESM.png]

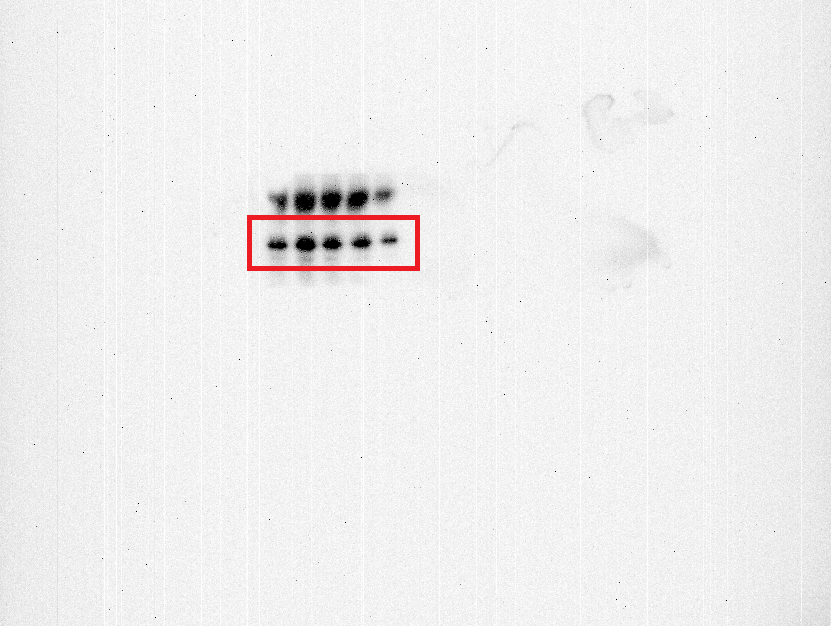

Supplement: Supplementary file 13 — Supplementary file13 (PNG 536 KB) [file 10753_2021_1586_MOESM13_ESM.png]

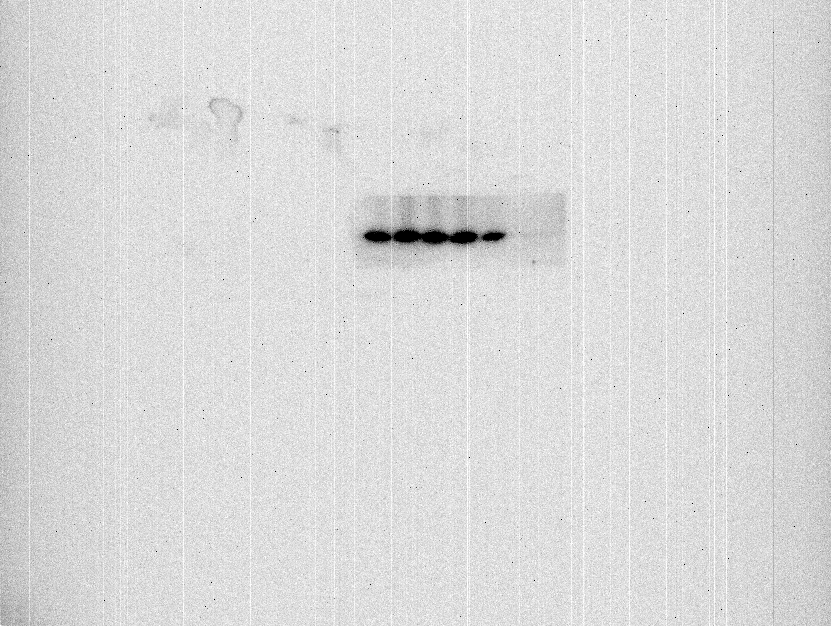

Supplement: Supplementary file 14 — Supplementary file14 (PNG 1527 KB) [file 10753_2021_1586_MOESM14_ESM.png]
